# Multiple stab wounds on the left side of the chest in a patient with Situs Inversus Totalis: A lifesaving coincidence

**DOI:** 10.1016/j.ijscr.2020.05.088

**Published:** 2020-06-12

**Authors:** Ismael Escobar Capriata, Caique Martins Pereira Ternes, João Vítor Ternes Rech, Franciele Kuhn Mesacasa, Robson Amaral, Orli Franzon

**Affiliations:** a Hospital Regional de São José Dr. Homero de Miranda Gomes, Department of Surgery, R. Adolfo Donato da Silva, s/n - Praia Comprida, São José, SC, 88103-901, Brazil; b Federal University of Santa Catarina Medical School, R. Eng. Agronômico Andrei Cristian Ferreira, s/n - Trindade, Florianópolis, SC, 88040-900, Brazil

**Keywords:** Situs inversus totalis, Stab wound, Emergency, Trauma, Cardiology

## Abstract

**Introduction:**

Situs Inversus Totalis (SIT) is a rare finding of complete reversal of the thoracic and abdominal organs with an estimated incidence of 0.005%–0.01% in the population. Severe trauma has not been reported in this population. We present a case of multiple chest stab wounds in a patient with previously unknown SIT.

**Presentation of case:**

A 39-year-old male was admitted to the emergency room with multiple stab wounds on the left side of the chest. Upon admission the patient was hypotensive, with miosis and intubated. Significant ECG findings were an inverted P wave, inverted QRS complex and inverted T wave in V1. A chest CT scan showed SIT, hemopneumothorax on the left side and, despite multiple stab wounds on the left side of the chest, no cardiac damage. The surgical team decided for a conservative approach and the patient remained in the ICU for two days. After five days he was discharged in good clinical conditions.

**Discussion:**

SIT generally does not have a clinical relevance throughout the patients life and most diagnoses are coincidental. However, when discovered in acute surgical cases, it requires an accurate evaluation by the surgical team due to anatomical differences that may produce undesirable outcomes in emergency cases such as appendicitis and general trauma.

**Conclusion:**

There have been very few reports of SIT and trauma in the medical literature. This might be the first ever reported case of a patient with SIT who suffered multiple stab wounds on the left side of the chest and was saved because of his condition.

## Highlights

• Reports of surgery in patients with SIT.
• Outcomes of emergency surgeries in patients with SituS Inversus Totalis can be different than those expected in the general population due to anatomical differences.

## 1 Introduction

Situs inversus totalis (SIT) is a rare condition characterized by a complete reversal of the anatomy of the thoracic and abdominal organs. It is a congenital autosomal recessive disease related to a X-chromosome defect [1]. The incidence of SIT is approximately 0.005%–0.01% [2]. Situs inversus totalis does not present with major implications for the patient and the diagnosis is usually coincidental. Although, in cases of trauma or other surgical emergency intervention, such as appendicitis, SIT may be an unexpected finding that needs to be considered by the surgical team [3,4]. There have been very few reports of SIT and trauma in the medical literature. We present a case compliant with the SCARE guidelines of a patient with Situs Inversus Totalis managed at the community Hospital Regional Dr. Homero de Miranda Gomes, São José - SC, Brazil, who suffered multiple stab wounds on the left side of the chest [5].

## 2 Presentation of case

**Fig. 1:**
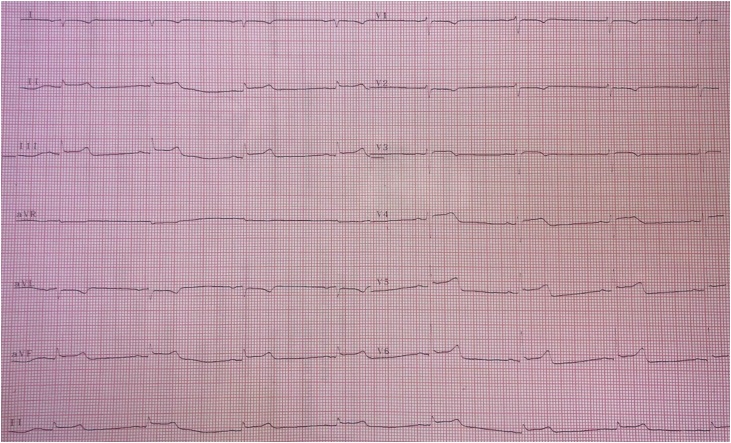
Electrocardiogram performed on admission evidencing right axis derivation, reverse precordial R-wave progression and an inverted P wave, QRS complex and T wave in V1.

A 39-year-old male patient was brought to the emergency room by the paramedics with multiple stab wounds on the left side of the chest, back and right lower limb. The patient reported to have used cocaine recently and had to undergo endotracheal intubation by the paramedics due to progressive desaturation on the way to the Emergency Room (ER). Upon admission the patient was hypotensive, with a Glasgow Coma Scale (GCS) of 3 and miosis on the neurological exam. The patient was given vasoactive drugs and volume reposition, with a positive response and the vital signs became stable. An electrocardiogram showed an inverted P wave, inverted QRS complex and inverted T wave in V1 (Fig. 1).

**Fig. 2:**
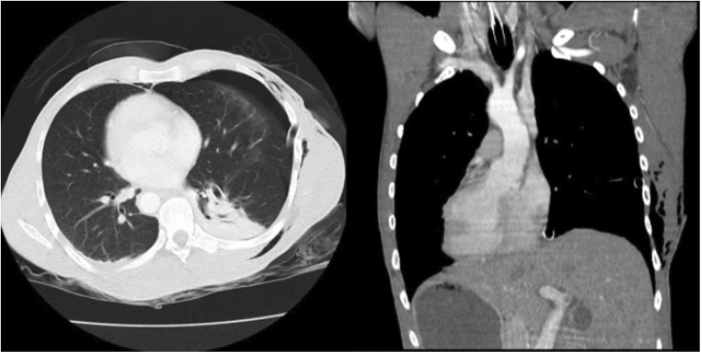
**CT scan findings.** A - Chest CT scan showing a hemopneumothorax to the left; B - Chest CT scan findings with dextrocardia and mirror-image transposition of the organs in the abdomen, confirming Situs inversus totalis.

A CT scan was performed to evaluate the extent of the injuries and revealed Situs inversus totalis, a left side hemopneumothorax and, despite the multiple stab wounds on the cardiac region, no cardiac damage was found (Fig. 2). The focused assessment with sonography for trauma (FAST) was negative. A chest tube was placed on the left side of the chest. The patient did not need further surgical interventions and was put on supplemental oxygen on the second day after admission. After five days the patient was discharged with antibiotics and in good clinical conditions.

## 3 Discussion

Situs inversus totalis (SIT) is a rare condition in which the organs lie on the opposite site of the expected common anatomy, frequently associated with Kartagener's syndrome, with no frequent clinical implications – usually found incidentally when the patient must undergo a surgical procedure or in cases such as the one reported, with trauma or emergency procedures [6]. Not many surgical cases in patients with SIT have been reported on medical literature, most of them related to oncological surgeries or appendicectomies, and only a few related to trauma [7,8].

In the cases of severe trauma that requires evaluation for a surgical intervention, the CT scan is the image exam of choice and an important tool for SIT diagnosis, even though some other exams may also be useful for the evaluation on the ER (such as electrocardiogram and FAST).

Penetrating trauma on the left side of the chest is a cause of myocardial injuries and vascular damage, and presents with a high mortality rate, often requiring urgent surgical intervention or at least a diagnostic laparoscopy for further elucidation of the lesions. This case, however, exhibited a very unlikely finding that probably spared the patient’s life. The multiple stab wounds on the typical cardiac region curiously resulted in only a few pulmonar concussions due anatomical variance of SIT.

## 4 Conclusion

Cases of extensive trauma injuries in patients with SIT are extremely rare. This might be the first ever reported case of a patient who suffered attempted murder with multiple stab wounds on the left side of the chest that would most likely have deceased if wasn’t for the dextrocardia associated with Situs inversus totalis. The patient presented, instead, with a few pulmonary concussions that were treated conservatively and he was discharged within a few days in good clinical conditions.

## Declaration of Competing Interest

Authors declare no conflict of interest.

## Funding

This research was not funded by any private or public entities.

## Ethical approval

Our paper is exempt from approval from an Ethics Commission because it’s a case report. Authorities of our institution were aware of this research and approved its submission for publication.

## Consent

Consent was obtained from the patient and documented in a signed consent form.

## Author contribution

Ismael E Capriata – Study design.

Caique M P Ternes – Data collection, analysis and writing.

João Vítor T Rech – Writing, editing and review.

Franciele K Mesacasa – Consulting.

Robson Amaral & Orli Franzon - Surgery department chiefs responsible for the assessment and management of this patient, overseeing and authorizing the publication of this case report.

## Registration of research studies

1. Name of the registry: This is not a “first-in-Man” study
2. Unique identifying number or registration ID:
3. Hyperlink to your specific registration (must be publicly accessible and will be checked):

## Guarantor

Ismael E Capriata.

## Provenance and peer review

Not commissioned, externally peer-reviewed.
